# Supplementary material for: Optimizing the Energy Consumption of Spiking Neural Networks for Neuromorphic Applications
Source: Front Neurosci. 2020 Jun 30;14:662. doi: 10.3389/fnins.2020.00662 (PMC7339957; doi:10.3389/fnins.2020.00662)
Supplement: Supplementary file 1 [file Image_1.PDF]

Optimizing the energy consumption of spiking neural networks for neuromorphic applications: Supplementary material

Martino Sorbaro, Qian Liu, Massimo Bortone and Sadique Sheik

Supplementary figure 1

This figure includes the results of additional experiments, which use a larger network (VGG16, [2]), starting from a pretrained model, fine-tuned using the SynOp loss. The task is a more difficult image recognition problem, on a 10-class subset of ImageNet called *ImageNette* [1], which uses much larger images compared to CIFAR10. We also ran these experiments using a simple L_1 penalty on activations, without per-layer weighting based on layer fanout. We draw three conclusions:

1. SynOp loss training can be effectively used as a final training step on pre-trained networks, removing the need for training from scratch.
2. The methods we illustrate are still effective when dealing with more difficult tasks and larger networks.
3. There is no evidence that fanout-weighting is necessary, as an unweighted L_1 penalty seems to lead to similar results, at least in this limited test. Weighting the penalty according to layer fanout is still our preferred choice, since it is a more direct proxy of power consumption – and the results may be different in other cases not evaluated here.

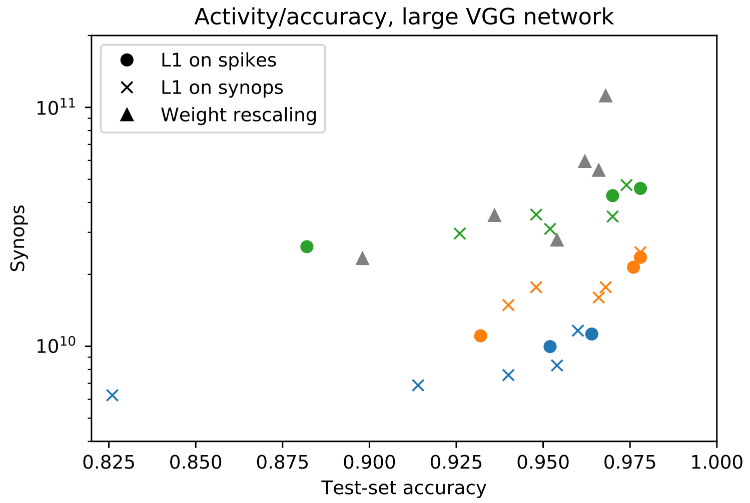

Figure 1: Activity/accuracy results for a VGG16 pre-trained network, fine-tuned using the SynOp loss. Colors correspond to different choices of rescaling of the input layer weights (causing increase or reduction of the overall activity). Crosses represent models trained with an L_1 penalty on the SynOp value (i.e. with fanout-weighting of the activations in the penalty). Circles represent models trained with a penalty on the number of spikes (non-weighted activations). Triangles are models trained with no additional loss term, for various values of input weight scaling.

Supplementary figure 2

In the SynOp loss penalty, we use an estimate of synaptic operations count, which is based on the quantized activations of the analog neural network. In this figure, we show that the value estimated in this way closely corresponds to the SynOp count actually observed in the simulated SNN with the same weights. Additionally there is also a good correspondence between the accuracy of the ANN and that of its associated SNN, limited to the regimes and networks used in this work. The models shown here are the same as those shown in figure 2 of the main text, with the same training methods, dataset and parameters.

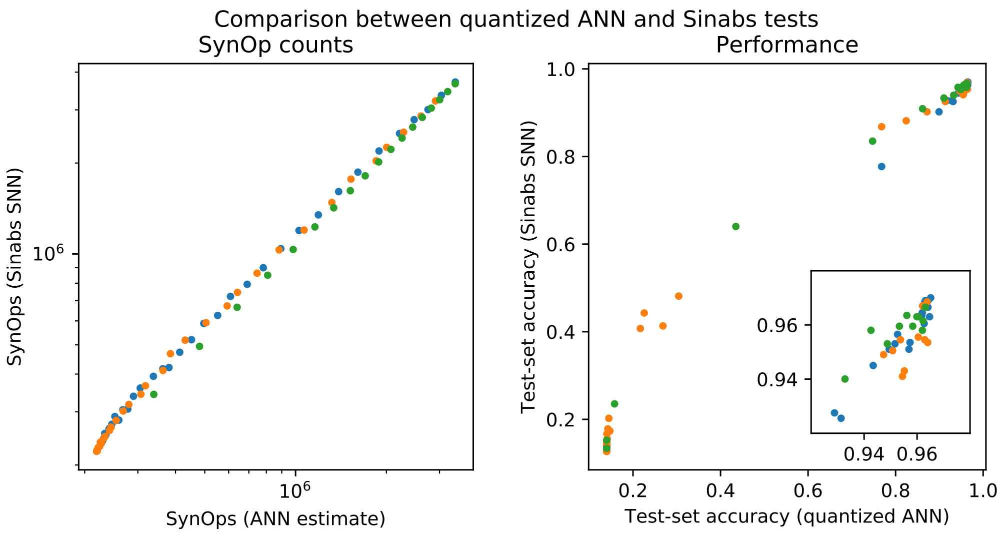

Figure 2: Left: comparison between the SynOps values as measured in our simulated spiking networks (from spike counts) and the SynOps estimated from the ANN (from quantized activations). Right: comparison between the accuracy of a quantized ANN versus the accuracy of the simulated SNN with the same weights. The inset shows the top-right area in more detail. The colors correspond to different training methods, with the same color scheme used in figure 2 of the main text.

References

[1] Jeremy Howard. Imagenette (https://github.com/fastai/imagenette), 2019.

[2] Karen Simonyan and Andrew Zisserman. Very deep convolutional networks for large-scale image recognition. *arXiv preprint arXiv:1409.1556*, 2014.
